# Microbiota and inflammatory bowel disease: the dual effect mechanism of polysaccharide therapy

**DOI:** 10.3389/fimmu.2025.1666866

**Published:** 2025-10-31

**Authors:** Jihao Yang, Qian Huang, Jingchun Long, Jia Li

**Affiliations:** ^1^ School of Acupuncture and Tuina, Guizhou University of Traditional Chinese Medicine, Gui’yang, China; ^2^ Department of Rehabilitation Medicine, Ji’an Central People’s Hospital, Ji’an, China

**Keywords:** inflammatory bowel disease, gut microbiota, polysaccharides, nanoparticles, gut metabolites

## Abstract

Inflammatory bowel disease (IBD) is characterized by chronic intestinal inflammation, strongly influenced by gut microbiota dysbiosis, barrier dysfunction, and immune imbalance. Increasing evidence highlights natural polysaccharides as promising therapeutic agents due to their dual roles in microbiota modulation and barrier reinforcement. Polysaccharides promote the growth of beneficial bacteria such as *Lactobacillus* and *Bifidobacterium*, enhance short-chain fatty acid (SCFA) production, and repair mucosal damage by upregulating goblet cells and tight junction proteins. These effects collectively restore microbial homeostasis and attenuate inflammation. Recent advances in polysaccharide-based nanocarriers, including chitosan, alginate, and hyaluronic acid, further enhance efficacy by enabling mucoadhesion, stimuli-responsive release, and targeted delivery within the inflamed colon. Such systems improve local drug retention, reshape the gut microenvironment, and amplify the therapeutic functions of polysaccharides. This review summarizes the pathological mechanisms of IBD, the regulatory effects of polysaccharides on gut microbiota, and the emerging role of nanotechnology in optimizing their delivery. Despite encouraging preclinical evidence, challenges remain regarding structural complexity, bioavailability, and clinical translation. Clarifying structure–activity relationships and developing multi-responsive nanocarriers represent future directions. Collectively, polysaccharides and their nanoformulations hold strong potential as safe and effective strategies for IBD therapy.

## 1 Introd*uc*tion

Inflammatory bowel disease (IBD) comprises chronic inflammatory disorders of the gastrointestinal tract, primarily Crohn’s disease (CD) and ulcerative colitis (UC). Dysbiosis of the gut microbiota is now recognized as a critical factor in both the initiation and progression of IBD (1). The intestinal microbiome, which includes bacteria, viruses, fungi, and parasites, contains approximately 100 trillion microorganisms (2). Consequently, therapeutic strategies aimed at restoring microbial balance have emerged as promising approaches for IBD management (3).

5-Aminosalicylic acid (5-ASA) remains a cornerstone of IBD therapy (4, 5). However, its use is associated with a spectrum of adverse effects, including headaches, nausea, hepatotoxicity, pancreatitis, bone marrow suppression, and renal complications (6). Prolonged treatment may also result in diarrhea, alopecia, and myalgia. Similarly, corticosteroids, although effective, are linked to risks such as osteoporosis, hypertension, obesity, type 2 diabetes, and exacerbation of gastrointestinal ulcers (7–9). Some of these drug-induced adverse reactions may even be life-threatening (10). In addition, while antibiotics can temporarily reduce pathogenic bacteria, long-term use disrupts microbial homeostasis and promotes resistance (11).

Natural polysaccharides have demonstrated considerable benefits in IBD treatment, as they reduce drug-related side effects while enhancing therapeutic efficacy (12–14). This reduction in drug-related side effects is closely linked to their regulatory role in gut microbiota: Evidence suggests that polysaccharides stimulate the growth of beneficial bacteria, facilitate intestinal barrier repair, and regulate gut metabolism by promoting the production of SCFAs—for instance, SCFAs can alleviate 5-ASA-induced intestinal mucosal damage by enhancing epithelial barrier repair, and mitigate corticosteroid-associated oxidative stress via activating antioxidant pathways (15). Moreover, specific microbial enzymes degrade polysaccharides, converting them into SCFAs and other secondary metabolites with health-promoting properties (16). Such processes regulate microbial communities and support intestinal homeostasis. Recent findings further indicate that polysaccharides improve barrier integrity, modulate the gut microenvironment, and enhance metabolic functions in patients with IBD (17).

Additionally, polysaccharide-based nanoparticles have attracted attention for their ability to improve drug stability and sustain therapeutic activity (18, 19). Given their dual ability to regulate gut microbiota and reinforce intestinal barrier function, polysaccharides represent a highly promising strategy for IBD therapy (20). This review systematically discusses the mechanistic basis of polysaccharide interventions and highlights their potential in future therapeutic development.

## 2 The relationship between the intestinal microbiome and IBD

Numerous studies have documented significant alterations in the intestinal microbiota of patients with IBD compared with healthy individuals (21–23). In healthy hosts, a dynamic balance is maintained among beneficial, potentially harmful, and commensal microbial populations (24). However, reductions in microbial diversity, shifts in metabolite profiles, disruption of the mucosal barrier, or immune dysregulation—often caused by disease, pharmacotherapy (e.g., antibiotics, laxatives), or unhealthy diets—can collectively drive the onset and progression of IBD (3, 25–29).

For example, one study reported markedly reduced levels of anti-inflammatory taxa such as *Faecalibacterium prausnitzii*, *Bifidobacterium adolescentis*, and other beneficial species in CD patients, alongside a significant increase in the pro-inflammatory species *Ruminococcus gnavus* (30). Similarly, murine models of colitis showed elevated levels of pathogenic genera including *Shigella*, *Aeromonas*, *Clostridium*, *Sutterella*, and *Akkermansia muciniphila*. These findings emphasize the contrasting roles of pathogenic and commensal bacteria in IBD pathophysiology (31, 32). Importantly, the impact of these bacteria is modulated by host immune status, environmental factors, and nutrient availability—conditions under which beneficial microbes may even exert neutral or deleterious effects, and vice versa.

Dynamic fluctuations in microbial populations critically influence gut diversity and ecosystem stability. In IBD, chronic inflammation disrupts this stability—pathogenic bacteria (e.g., *Ruminococcus gnavus*) proliferate rapidly, while beneficial taxa (e.g., *Faecalibacterium prausnitzii*) decline, leading to chaotic microbial fluctuations and reduced diversity (30, 32). Probiotic intervention can counteract such disruptive fluctuations: In a study of DSS-induced colitis, administration of *Lactobacillus rhamnosus* GG (LGG, 10^9^ CFU/day) markedly improved microbiota diversity, reversing dysbiosis by enriching beneficial taxa such as Bifidobacterium, *Olsenella, Paenibacillus*, and *butyrate-producing bacteria* (33). Moreover, a 14-day LGG intervention suppressed pathogenic clusters, including Escherichia coli, *Zhiphyllobacterium, Osteobacillus*, and *Desulphurobacteria*, thereby alleviating colonic inflammation in UC mouse models (34).

Under physiological conditions, the mucus layer segregates luminal bacteria from intestinal epithelial cells (IECs), and the immune system maintains tolerance toward luminal antigens (26, 35). In IBD, barrier disruption increases permeability, enabling bacteria to contact IECs directly (36) and translocate into systemic circulation (37). This translocation triggers inflammatory cytokine expression and immune activation (38, 39). Barrier dysfunction is further characterized by reduced mucin content (40), diminished glucose-derived metabolites (41), impaired lipid-associated protective factors (42), and decreased secretion of pancreatic-derived defense molecules (43).

Metabolic studies further link microbial composition to functional pathways. Dysbiotic states are associated with reduced polysaccharide-degrading capacity and upregulated oxidative stress-related genes (44). Excess bacterial metabolites elevate ROS, thereby exacerbating epithelial injury and inflammation (45). Elevated *Desulfovibrio* spp. in IBD patients promote hydrogen sulfide overproduction, inducing oxidative stress, damaging IECs, and aggravating mucosal inflammation (46).

Additionally, dysbiosis frequently results in SCFA depletion, which worsens intestinal inflammation (47–49). As major products of polysaccharide fermentation, SCFAs suppress pathogen proliferation, enhance nutrient absorption, regulate immune responses, and reinforce the mucus barrier (50–53). They lower intestinal pH, facilitating the colonization of beneficial bacteria such as *Lactobacillus* and *Bifidobacterium*. These bacteria further ferment carbohydrates into SCFAs, strengthening mucosal immunity (54). Probiotics such as *Bifidobacterium* and *Lactobacillus* mitigate inflammation by modulating NF-κB signaling, enhancing epithelial adhesion, and inhibiting pathogens (55, 56). The dominant gut phyla, *Bacteroidetes* and *Firmicutes*, produce acetate and propionate (mainly *Bacteroidetes*) and butyrate (predominantly *Firmicutes*) (57). Butyrate, in particular, serves as the primary energy source for IECs and promotes epithelial proliferation, which is essential for mucosal repair (58).

## 3 Disruption of the intestinal upper barrier and imbalance of the microbiome

### 3.1 Interaction between the intestinal microbiome and the mucous layer

The intestinal surface is covered by a bi-layered mucus structure that plays a fundamental role in preserving the integrity of the upper intestinal barrier (59). The outer mucus layer directly interfaces with the gut microbiota and provides a nutrient source for commensal species. Certain bacteria, such as *Akkermansia muciniphila* and *Bacteroides fragilis*, secrete mucin-degrading enzymes that remodel this layer to facilitate colonization. A healthy microbiota also contributes to the maturation of gut-associated lymphoid tissue and modulates immune responses, thereby preventing pathogen invasion and endotoxin translocation. In addition, commensal microbes reinforce mucus barrier function by reducing luminal oxygen levels and stimulating host immune activity. Microbial metabolites act as key molecular mediators, directly regulating mucosal immune signaling, shaping host physiology, and maintaining immune homeostasis.

Johansson et al. demonstrated that *Erysipelotrichia* and *Paenibacillus* decreased mucus permeability, whereas *Proteobacteria* and *Saccharibacteria* increased it, highlighting that mucus properties are strongly influenced by microbiota composition (60). Probiotic supplementation can also upregulate mucin synthesis. For example, lymphocyte-associated pathways induce *Mucin 2* expression in IECs, while colonization by rod-shaped bacteria restores mucus production (61). These findings underscore the impact of probiotics on mucus barrier integrity and intestinal health (62, 63).

Animal studies further revealed a marked reduction in goblet cells in germ-free mice (64). The mucus layer in these animals lacked critical immune molecules, such as regenerating islet-derived protein III, rendering them more vulnerable to bacterial infection. Under severe infection, the intestinal mucosa compensates by secreting large amounts of mucus to physically limit bacterial invasion (65). Moreover, SCFAs—key metabolites of the gut microbiota—regulate mucus dynamics, stimulating mucin secretion at low concentrations but potentially suppressing it at higher levels (66–68).

### 3.2 Influence of microbiota on IECs

The renewal and coordinated function of IECs are essential for maintaining barrier integrity. Together with gut microbes and the mucosal immune system, IECs constitute the first line of defense against luminal pathogens and antigens (69). Exposed directly to the intestinal lumen, IECs express a wide array of pattern recognition receptors that enable microbial sensing. Beneficial microbes form a protective biological barrier on the mucosal surface, outcompeting pathogens for adhesion sites, secreting antimicrobial compounds, stimulating mucus production, and strengthening tight junction complexes. Collectively, these interactions promote IEC growth, regeneration, proliferation, and repair, thereby preserving mucosal barrier function (70–73).

In contrast, pathogenic colonization disrupts commensal communities and adversely affects IEC structure and function (74). In IBD patients, elevated populations of sulfate-reducing bacteria produce hydrogen sulfide, which damages IECs and triggers mucosal inflammation (75). Notably, such pathogenic colonization also inhibits the growth of SCFA-producing bacteria (e.g., *Firmicutes*), reducing SCFA availability—a double blow to IEC homeostasis. Conversely, SCFAs, as key microbial metabolites, can reverse IEC damage: SCFAs interact with G-protein-coupled receptors (GPCR41 and GPCR43) on IECs to induce enteroendocrine hormone release, thereby modulating disease processes (76). Notably, butyrate provides a major energy source for IECs, regulates cell proliferation and differentiation, and stimulates Paneth cells to secrete antimicrobial peptides through GPCR43 signaling (77). Other microbial metabolites, including succinate and propionate, also contribute to IEC growth, differentiation, and colonic energy metabolism (78).

In addition, epithelial polysaccharides serve a decisive role in shaping gut microbiota by providing binding ligands and nutritional substrates, thereby influencing microbial composition and colonization. Recent evidence links IBD to altered O-glycan expression in the mucus layer, including increased levels of short-chain O-glycans and modified terminal structures. These changes impair mucus barrier function, disrupt lectin-sugar interactions, disturb host-microbe communication, and weaken mucosal immunity, collectively promoting IBD pathogenesis (79).

### 3.3 Effect of gut microbiota on intestinal permeability

The preceding discussion on microbial interactions with the mucus layer and IECs underscores how the gut microbiota governs multiple aspects of barrier function. To further clarify how specific bacterial species regulate barrier integrity—particularly mechanisms that alter intestinal permeability—additional analysis is warranted.

A prominent example is adherent-invasive *Escherichia coli* (AIEC), which has been closely associated with intestinal inflammation. Patients with IBD often exhibit elevated AIEC abundance. These bacteria compromise barrier function by directly increasing intestinal permeability, disrupting microbial diversity, and modulating the expression of inflammatory mediators (80). Enhanced colonization of epithelial-adherent pathogens such as AIEC exacerbates mucosal permeability, reshapes microbial community composition, and initiates inflammatory cascades via upregulation of pro-inflammatory genes, ultimately driving intestinal inflammation (81).

Conversely, certain probiotic strains protect against barrier disruption. *Escherichia coli* Nissle 1917 (EcN), a Gram-negative, non-lactic acid probiotic strain, can colonize the gut stably, interact with IECs and resident microbes, and exert protective effects. In T84 colonic epithelial cell models, Zyrek et al. demonstrated that EcN upregulates tight junction proteins zonula occludens-1 (ZO-1) and zonula occludens-2 (ZO-2), thereby preserving mucosal integrity and reducing permeability (82). In murine studies, oral administration of EcN (10^9^ CFU/day) alleviated colitis symptoms, improved histopathological outcomes, protected intestinal permeability, reduced neutrophil and eosinophil infiltration, decreased chemokine and cytokine levels, and increased regulatory T cell populations within Peyer’s patches (83).

Mechanistically, EcN’s ability to preserve tight junction architecture is thought to involve the MLCK/MLC signaling pathway. Further investigations into EcN-mediated regulation of tight junction proteins—such as ZO-1, claudin-1, and occludin—through MLCK pathway modulation may provide deeper insights into molecular mechanisms underlying its barrier-protective effects.

## 4 intestinal microecology and intestinal immunity

**Figure 1:**
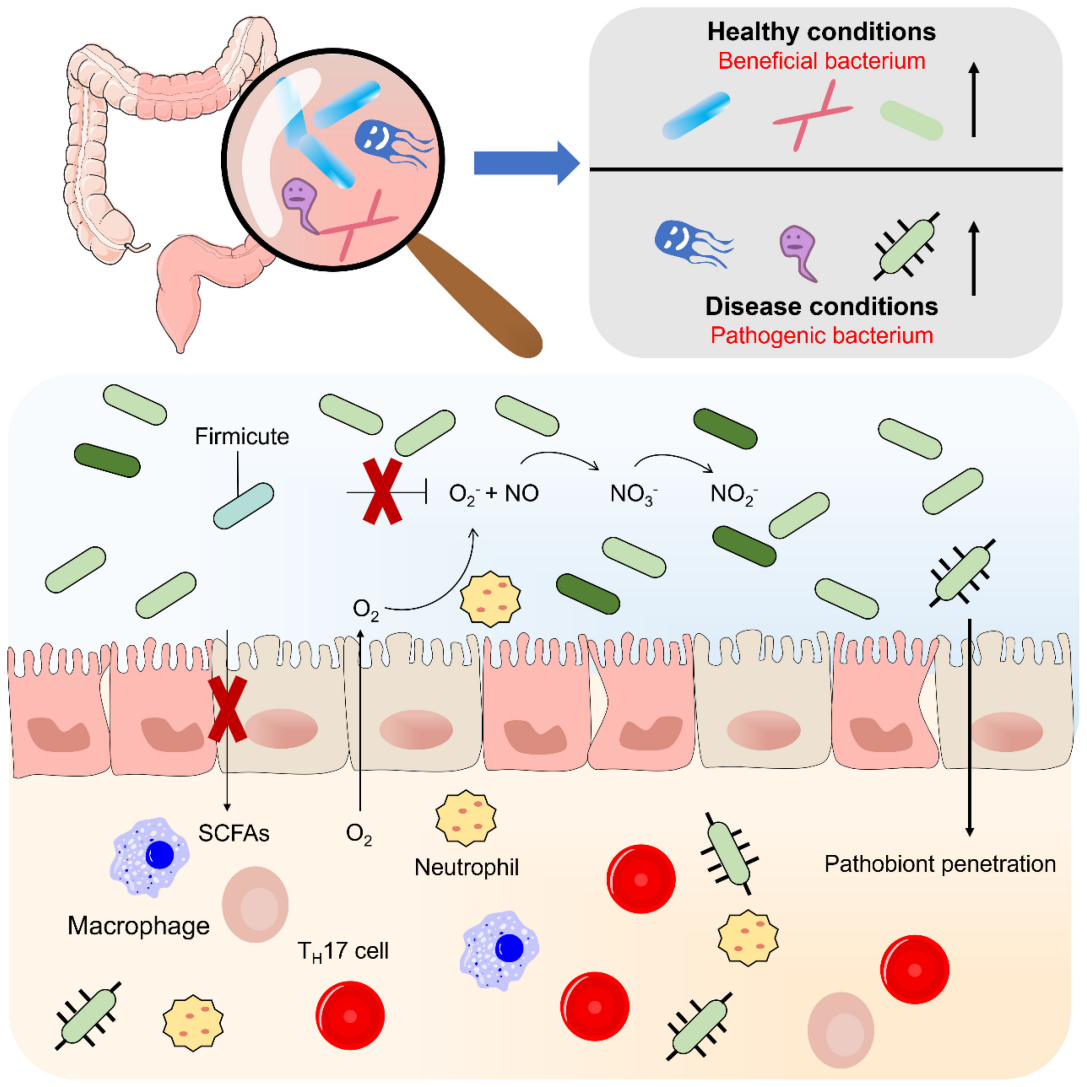
Flora disorders in inflammatory bowel disease. According to their relationship to the human body, normal intestinal bacteria can be divided into three categories: 1 symbiotic (beneficial) 2 conditional (neutral) 3 pathogenic (harmful). Inflammation leads to changes in bacterial clusters, such as the proliferation of deformed bacteria, caused by increased oxygen synthesis in the inflammatory intestinal environment, nitrate (NO-) and increased availability of host-generated oxygeon receptors and iron. The flora disorder is manifested by a general decrease in microbial diversity and a loss of beneficial bacteria, which may lead to increased mucous adhesion and transfer of the beneficial flora.

The inflammatory response in IBD originates from the activation of innate immune cells—including macrophages, dendritic cells, neutrophils, natural killer cells, and innate lymphoid cells—which release cytokines, chemokines, and antimicrobial peptides. This innate activation subsequently triggers adaptive immunity, with T and B lymphocytes serving as central mediators of intestinal inflammation in IBD (84) (**Figure 1**).

Accumulating evidence indicates that distinct microbial communities differentially regulate T cell-mediated immunity. For instance, *Faecalibacterium prausnitzii* alleviates chemically induced colitis in mice by enhancing regulatory T cell (Treg) activity (85). Similarly, *Clostridium butyricum* promotes the expansion of CD4^+^Foxp3^+^ Tregs in the intestinal lamina propria, thereby preventing colitis and attenuating hypersensitivity reactions (86).

Invariant natural killer T (iNKT) cells, which share features of both NK and T cell lineages, also contribute to intestinal immunoregulation. Germ-free mice display heightened susceptibility to iNKT-mediated colitis and asthma following oxazolone or ovalbumin challenge. This susceptibility arises because germ-free conditions lack microbial metabolites (e.g., SCFAs, bile acid derivatives) that suppress iNKT cell overactivation—neonatal exposure to commensal microbes promotes the production of these metabolites, thereby limiting iNKT-mediated inflammatory responses. Remarkably, neonatal exposure to commensal microbes is required to mitigate this susceptibility (87).

During programmed cell death, neutrophils release neutrophil extracellular traps (NETs)—web-like structures composed of chromatin, histones, proteases, granule proteins, and enzymes such as myeloperoxidase and neutrophil elastase. NETs restrict pathogen dissemination and exhibit bactericidal activity through associated proteases. However, their components can also disrupt immune homeostasis (88). NETs have been shown to influence B cell differentiation and function. In rheumatoid arthritis, for example, NET-immune cell interactions promote B cell proliferation and autoantibody production via B cell-activating factor, while synovial NETs provide citrullinated proteins that fuel anti-citrullinated protein antibody (ACPA) responses (89).

In IBD, depletion of SCFA-producing bacteria—particularly butyrate producers—correlates with increased neutrophil infiltration and NET formation, thereby accelerating disease progression (90). Microbial metabolites, especially SCFAs, also shape macrophage function: SCFAs suppress pro-inflammatory cytokine production by inhibiting histone deacetylases, while upregulating anti-inflammatory IL-10 (91). Butyrate, in particular, inhibits HDAC3, reduces mTOR activation and glycolysis, enhances macrophage bactericidal activity, and promotes an anti-inflammatory phenotype (92).

Considerable evidence supports a role for SCFAs in regulating CD4^+^ T cell subsets, particularly Tregs, which are critical for immune tolerance. SCFAs promote Treg differentiation by inhibiting HDAC activity; however, under strong anti-CD3 stimulation favoring Th1/Th17 polarization, this induction is attenuated (93). Although less studied, SCFAs also affect CD8^+^ T cells, which are essential for intracellular pathogen clearance and tumor surveillance. For example, systemic acetate elevation during bacterial infection enhances glycolysis and boosts memory CD8^+^ T cell recall responses (94).

Beneficial microbes such as *Bifidobacterium* and *Lactobacillus* further modulate intestinal Treg populations, highlighting their role in IBD pathogenesis (95). Treg-deficient mice spontaneously develop colitis, underscoring the indispensable role of Tregs in intestinal homeostasis (96). Moreover, LGG promotes B cell differentiation and IgA secretion in the intestinal lamina propria of piglets, thereby strengthening mucosal immunity (97).

Additional mechanisms involve microbial regulation of innate sensors and inflammatory pathways. For instance, *Bacteroides fragilis* suppresses NLRP3 inflammasome activation via SCFA production, thereby inhibiting M1 macrophage polarization and reducing pro-inflammatory cytokines such as IL-18 and IL-1β (98). *Faecalibacterium prausnitzii* exerts anti-inflammatory effects by downregulating IL-12 and IFN-α, while enhancing IL-10 secretion and inhibiting NF-κB signaling (85, 99).

## 5 Gut microbiota and oxidative stress

In IBD, excessive production of ROS—including superoxide anions, peroxynitrite, hypochlorite, and hydrogen peroxide—has been strongly implicated in disease progression. ROS directly damage IECs, activate mucosal immune responses, and trigger oxidative stress-related signaling pathways such as NF-κB and nuclear factor erythroid 2-related factor 2 (Nrf2), thereby aggravating barrier dysfunction and chronic inflammation (100, 101).

Metagenomic analyses of IBD patients have revealed downregulation of genes involved in carbohydrate and amino acid metabolism, coupled with upregulation of genes associated with oxidative stress responses. These findings suggest that altered microbial metabolic capacity may exacerbate IBD by enhancing oxidative stress and impairing epithelial integrity.

LGG exhibits potent antioxidant activity and supports intestinal barrier function. LGG reduces oxidative stress-induced damage by enhancing endogenous antioxidant enzyme production, including superoxide dismutase (SOD) and glutathione (GSH), while simultaneously suppressing ROS generation. These protective effects are mediated through activation of the Keap1/Nrf2 pathway and inhibition of ERK1/2 and NF-κB signaling (102). Further studies demonstrate that LGG reduces *Giardia*-induced colonization, enhances antioxidant defenses, and lowers lipid peroxidation, thereby maintaining epithelial integrity (103).

In a hydrogen peroxide-induced oxidative stress model using porcine IECs, extracellular polysaccharides from LGG accelerated ROS clearance by upregulating antioxidant enzyme expression and downregulating oxidative stress-related proteins. These effects collectively facilitated the repair of epithelial barrier damage (104).

Additional studies have investigated synergistic effects of natural polysaccharides with bioactive plant-derived compounds. For example, *Lycium barbarum* polysaccharides (LBPs), known for immunomodulatory properties, and capsaicin, an anti-inflammatory and antioxidant agent, were tested in a DSS-induced colitis rat model. LBPs alone decreased serum IL-6 and malondialdehyde (MDA, a lipid peroxidation marker), while enhancing catalase activity. Co-administration of LBPs and capsaicin further reduced IL-6 and colonic tumor necrosis factor-α (TNF-α), and significantly increased SOD activity. These results underscore the synergistic antioxidant and anti-inflammatory potential of combining natural polysaccharides with plant bioactives in ulcerative colitis therapy (105).

## 6 Polysaccharides regulate the gut microbiota

**Figure 2:**
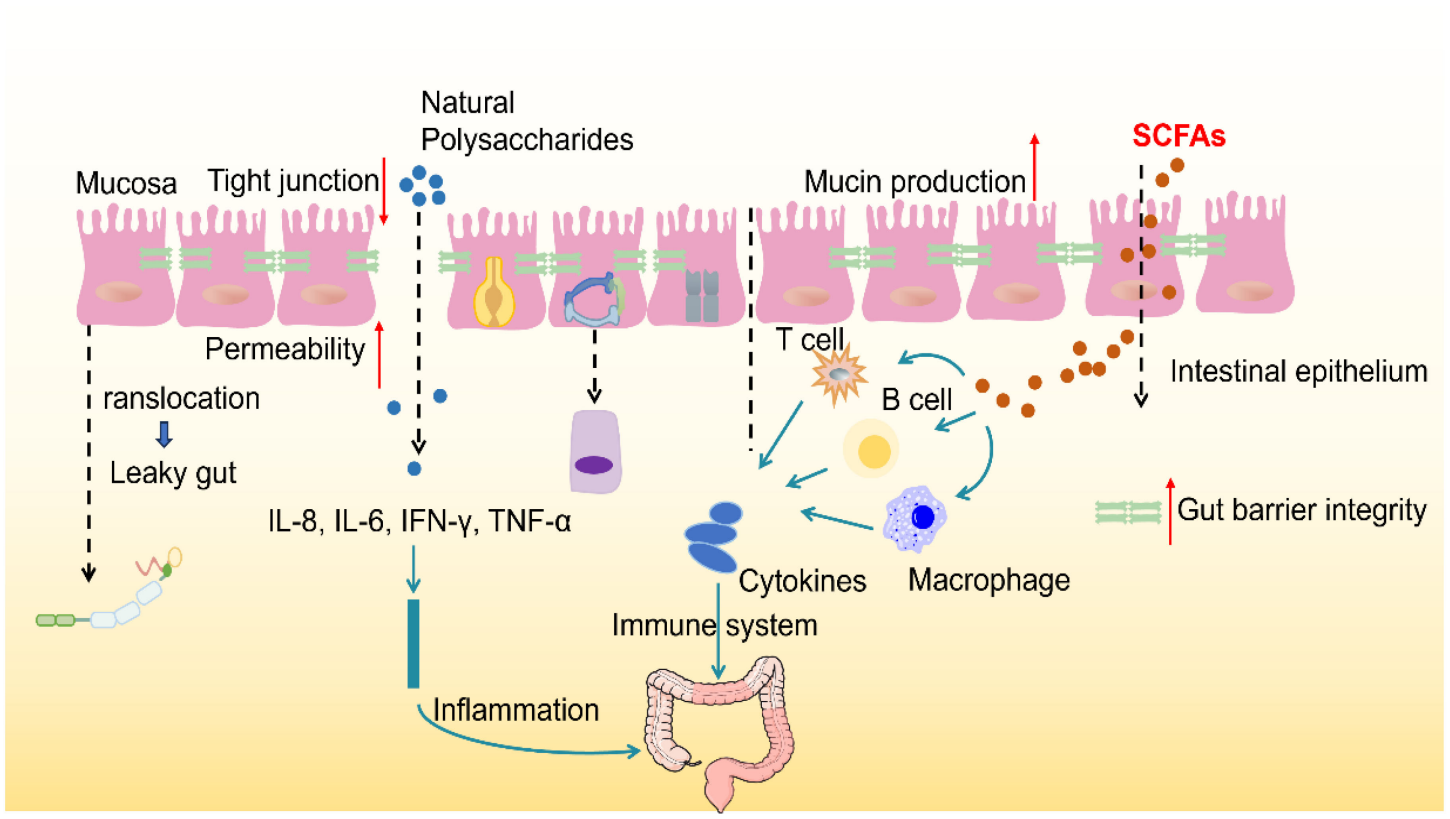
Polysaccharides modulate gut microbiota and enhance gut barrier function. The healthy gut barrier consists of a Tight layer of IECS, which are interconnected by Tight junctions that control the Permeability of matter. In good health, the intestinal immune system maintains the immune balance by secreting cytokines (such as IL-2, IL-5, IL-6, IL-9, TNF-α and IFN-γ) and activating immune cells (such as T cells, B cells and macrophages). Mucins, antimicrobial peptides and secretory IgA can form biochemical barrier and enhance intestinal protective function. Goblet cells secrete mucins that form a dense, sticky, and permeable gel that coats the intestinal mucosa, preventing erosion by microbes. Once the tight junctions are broken, a phenomenon known as Leaky gut is formed, which increases intestinal permeability and allows harmful substances and pathogens to enter the bloodstream.

Natural polysaccharides are widespread biological macromolecules that function as structural components, energy reserves, and protective agents in diverse organisms. They are complex carbohydrates formed by the condensation of multiple monosaccharide units through glycosidic bonds, generally represented by the formula (C_6_H_10_O_5_)_n_ (106). Polysaccharides can be derived from plants, algae, animals, or microorganisms (107, 108). Their physicochemical properties—including monosaccharide composition, chain length, branching degree, and substituents—profoundly influence their bioactivities. Hydrophilic groups such as hydroxyl, carboxyl, and amino groups confer high solubility and dispersibility, while additional functional groups permit chemical modification, enabling the formation of functionalized supramolecular structures (109) (**Figure 2**).

However, due to variations in monosaccharide composition, degree of polymerization, and linkage patterns, extraction and purification of polysaccharides are inherently complex. For example, polysaccharides with high branching degrees require more precise ethanol concentration adjustments to avoid co-precipitation with impurities; high-molecular-weight polysaccharides (due to high polymerization degrees) easily clog membrane pores during separation, necessitating stricter pressure control; and different monosaccharide linkages affect the binding affinity to macroporous resins, complicating elution gradient design. In plant polysaccharide extraction, ethanol precipitation is commonly applied to remove proteins, lipids, nucleic acids, pigments, and other small molecules from crude extracts, followed by fractionation to obtain homogeneous polysaccharides. Frequently used purification strategies include macroporous adsorption resins for initial separation, ion-exchange chromatography for selective fractionation, and membrane separation techniques exploiting molecular weight cut-offs under controlled pressure (110, 111).

Polysaccharides are generally classified by source into animal-, plant-, microbial-, and marine-derived types (112). Animal polysaccharides—often early pharmaceutical candidates—are typically mucopolysaccharides with high water solubility (113, 114). Plant-derived polysaccharides, such as pectin, *Angelica*, *LBP*, rhubarb, and *Bupleurum* polysaccharides, are usually water-soluble and low in toxicity, making them suitable for precise dosing in experimental settings (115). In contrast, starch and cellulose are insoluble plant polysaccharides. Microbial polysaccharides are produced by bacteria and fungi, while marine polysaccharides, isolated from aquatic organisms, often possess unique biological activities (116, 117).

Functionally, polysaccharides act as fermentable carbon sources for probiotics, promoting their growth, reshaping microbial community structure, and suppressing pathogenic bacteria (118). For example, polysaccharides extracted from *Polygonum multiflorum* increase populations of *Bifidobacterium* and *Lactobacillus* while decreasing *Helicobacter*, thereby alleviating gut dysbiosis and contributing to IBD management (119). In juvenile *Hucho taimen*, dietary supplementation with lentinan enhanced beneficial genera such as *Lactobacillus*, *Trichinella*, and *Ruminococcus*, while reducing harmful taxa including *Enterobacteriaceae*, *Fusobacteriaceae*, and *Flavobacteriaceae*, thus improving microbial balance (120).

The structural features of polysaccharides also determine preferential fermentation by specific microbes (121). For instance, oat β-glucans selectively promote *Bifidobacterium* and *Lactobacillus* (122), while *Bacteroides* efficiently degrade fructans (123), and *Prevotella bryantii* utilizes xylan (124). *In vitro* fermentation studies with Fuzhuan brick tea polysaccharides (FBTPS-3) showed modulation of IBD patient microbiota toward a profile resembling that of healthy individuals, specifically by increasing *Bacteroides* and decreasing *Escherichia/Shigella* (125).

Beyond shaping microbial composition, polysaccharides reinforce the mucus barrier by enhancing thickness, adhesiveness, and protective capacity, thereby preventing pathogen invasion (126). Dietary polysaccharides also directly upregulate tight junction proteins such as occludin and ZO-1, strengthening epithelial barrier integrity. For example, interventions significantly enhanced occludin and claudin-1 expression while reducing pro-inflammatory cytokines including TNF-α and IL-1β (127).

Polysaccharides from natural sources have demonstrated protective effects against colitis. *Gloiopeltis furcata* polysaccharides safeguard colonic mucosa by modulating mucin-microbe interactions, promoting probiotic growth, and reducing epithelial injury (128). *Dendrobium huoshanense* polysaccharides increase goblet cell numbers and stimulate mucin secretion in both small and large intestines, reinforcing mucosal defenses (129). Functionalized fucoidan restores microbial balance and mucosal integrity after injury (130). *In vitro* studies reveal that glucomannan from *Aloe vera gel* maintains barrier function via the Nrf2-mitochondrial axis and alleviates anoikis induced by mitochondrial dysfunction (131).

Marine polysaccharides such as fucoidan exhibit strong anti-inflammatory and immunomodulatory activity by inhibiting NF-κB signaling and downregulating TNF-α, IL-6, and IL-8 (132). Fucoidan also promotes Treg differentiation, enhances tight junction proteins and IgA secretion, and reduces intestinal permeability (133). In murine models, fucoidan mitigates colitis by lowering nitric oxide, myeloperoxidase, and malondialdehyde levels, reducing immune cell infiltration, and preserving colon length (134, 135). Additionally, fucoidan may stimulate dendritic cell maturation via TNF-dependent pathways, strengthening host immunity.

Other plant polysaccharides also improve IEC structure. Yam polysaccharides maintain epithelial morphology, increase goblet cell density, and decrease inflammatory infiltration (136). High-molecular-weight fucoidan from *Undaria pinnatifida* and *Sargassum fusiforme* protects Caco-2 cells against ROS-induced injury, likely via antioxidant activity (137). Composite polysaccharides (e.g., yam plus inulin) modulate microbiota by reducing *Proteus*, *Bacteroides*, and *Firmicutes*, enhancing metabolism, and relieving oxidative stress, ultimately improving ulcerative colitis (138).

Polysaccharide-mediated modulation of SCFA production represents another therapeutic mechanism (139). *Ganoderma lucidum* polysaccharides significantly increase acetate, propionate, and butyrate levels (140, 141). Similarly, *LBPs* undergo fermentation to generate SCFAs while enriching *Bifidobacterium* and *Lactobacillus* (142). Longan polysaccharides enrich SCFA-producing taxa such as *Bifidobacterium*, *Bacillus*, and *Bacteroides fragilis*, boosting acetate, propionate, and butyrate synthesis (143). Seaweed polysaccharides also act as fermentation substrates, indirectly supporting probiotic growth (144–147).

Importantly, the structural features of polysaccharides dictate fermentation kinetics and SCFA profiles, with identical fermentation conditions producing variable SCFA yields (148). Thus, targeted research on specific polysaccharides is required to define their optimal application in colitis therapy (**Table 1**). Taken together, natural polysaccharides reshape gut ecology (beneficial taxa↑, SCFAs↑), reinforce the epithelial barrier (mucus/TJ proteins↑), rebalance mucosal immunity (Tregs↑, NF-κB↓), and mitigate oxidative stress (Nrf2 axis↑). However, these biological benefits are highly contingent on local concentration and residence time at inflamed colonic sites. Oral administration faces substantial hurdles—acidic gastric milieu, digestive enzymes, rapid mucus clearance, and heterogeneous lesion distribution—leading to suboptimal on-target exposure. This translational gap motivates an engineering solution: polysaccharide-based nanomedicines that exploit the inflammatory microenvironment (pH↓, ROS↑, bacterial glycosidases↑, receptor overexpression) to achieve spatiotemporally controlled delivery and thereby amplify the very mechanisms delineated above.

**Table 1:** Polysaccharides regulate the gut microbiota.

Polysaccharides	Models	Gut microbiota regulation	References
Agaricus blazei Murrill polysaccharide	DSS-induced colitis in mice	*Ruminosae* and *Oxisella*↑;*Lactobacillus* and *Shigella*↓	(149)
Mytilus coruscus polysaccharide	DSS-induced colitis in mice	*Anaerotruncus Lactobacillus Alestris Odoles Desulphurization Vibrio* and *Intestinal vibrio↑;Bacteroides Longiella* and *Specific bacteria↓*	(150)
Astragalus polysaccharides	DSS-induced colitis in mice	*Lactobacillus casei Lactobacillus acidophilus Rhamnobacter Long yeast Saccharomyces cerevisiae Rhamnococcus acidophilus* and *Actinomycetes↑;Vibrio desulphurization Bifidobacterium Lactococcus Escherichia coli* and *Citrobacter↓*	(151)
Large Yellow Tea Polysaccharide	HFD-induced intestinal homeostasis dysbiosis in mice	*Ileibacterium Lactobacillus Bifidobacterium* and *Akkermansia↑;Dubosiella Faecalibaculum Coriobacteriaceae_UCG-002* and *Erysipelatoclostridium↓*	(152)
Pumpkin polysaccharides	DSS-induced colitis in mice	*Lactobacillus Culturing bacteria Slime* sp*irochetes Shigella Alistipes Helicobacteria* and *Campylobacter↑*	(153)
Callicarpa nudiflora Hook polysaccharides	DSS-induced colitis in mice	*Desulfovibrio Clostridium_XlVa Flavonifractor Barnesiella Oscillibacter Pseudoflavonifractor Clostridium_IV Firmicutes Proteus Tropomycetes* and *Verruca↑;Bacteroidetes Bdelloides Bacteroidetes* and *Proteus↓*	(154)
Schisandra chinensis (Turcz.) Baill. polysaccharide	DSS-induced colitis in mice	*Baculaceae* and *Lachnospiraceae_NK4A136↑;Bacteroides* and *Erysipelatoclostridium↓*	(155)
Pectic polysaccharides from Aconitum carmichaelii leaves	DSS-induced colitis in mice	*Acinetobacter A. finegoldii Prevotella 9* and *Lachnospira↑;Bacteroides Alistipes Streptococcus Ruminococcus* and *Dubosiella↓*	(156)
Bamboo (Phyllostachys edulis) shoot polysaccharide	DSS-induced colitis in mice	*Prevotella Aliti Anaerobes Stenobacteria Bifidobacterium Butyrobacter* and *Lactobacillus↑;Lactobacillus parasitosus Slime* sp*irochetes Helicobacteria* and *Streptococcus↓*	(157)
Floral mushroom polysaccharide	DSS-induced colitis in mice	*Lachnospiraceae_NK4A13G Odoribacter↑;Bacteroides Helicobacter* and *Parasutterella↓*	(158)
Allium tenuissimum L. flowers polysaccharide	DSS-induced colitis in mice	*Lachnospiraceae* and *Alloprevotella↑; Bacteroides Lactobacillus Pneumococcus Anaerovoracaceae* and *Butyricicoccaceae↓*	(159)
Laiyang pear residue polysaccharides	DSS-induced colitis in mice	*Actinomycetes* and *Lactobacillus↑; Verrucomicrobiota Turicibacter* and *Romboutsia ↓*	(160)
Safflower polysaccharide	DSS-induced colitis in mice	*Verrucomicrobiota* and *Akkermansia↑;Bacteroides↓*	(161)
Rosa laevigata polysaccharides	DSS-induced colitis in beagles	*Prevotella Bacteroides Faecalibacterium Turicibacter Toricibacter* and *Megamonas↑;Romboutsia* and *Terrisporobacter↓*	(162)
Ishige okamurae polysaccharide	DSS-induced ulcerative colitis in mice	*Bacteroidetes Campylobacter and Proteus↑; sessile fungi Actinomycetes Dubosiella Romboutsia norank_f_norank_Clostridia_UCG-014 Bifi-dobacterium Coriobacteriaceae_UCG-002 Saccharimonas Allobaculum unclassified_f_Prevotellaceae* and *Tyzzerella↓*	(163)
Sagittaria sagittifolia L. polysaccharides	DSS-induced colitis in mice	*Firmi- cutes Bacteroidetes Lactobacillus* and *Yeasts↑;Aspergillus ligilactobacillus* and *Akkermansia↓*	(164)
Gastrodia elata polysaccharides	DSS-induced colitis in mice	*Eosinophilic bacteria Ligilactobacillus* and *Alloprevotella↑;Bacteroides* and *EscherichiaShigella↓*	(165)
Tamarind seed polysaccharide hydrolysate	DSS-induced ulcerative colitis in mice	*Akkermansia Prevotella* and *Blautia↑; Coprobacillus↓*	(166)
Sea buckthorn polysaccharide	DSS-induced colitis in mice	*Prevotella Prevotella Allobaculum Escherichia Saudi Clostridium Parabacteroides* and *Escherichia ↓*	(167)
Rehmannia glutinosa polysaccharide	DSS-induced colitis in mice	*Solid condensation bacteria Lactobacillus Alistipes* and *Lachnospiraceae_NK4A13↑;Bacteroides* and *Proteobacteria↓*	(168)
Nostoc commune Vaucher polysaccharide	DSS-induced acute ulcerative colitis in mice	*Akkermansia muciniphila g norank_f Muribaculaceae* and *g norank_f norank_o Clostridia_UCG-014↑*	(169)
Paecilomyces hepiali polysaccharides	DSS-induced colitis in mice	*Bacteroides* and *Desulfobacterota↑;Firmicutes Verrucomicrobiota Deferribacterota Desulfovibrionaceae Anaerovoracaceae Oscillospiraceae Enterobacteriaceae* and *Lachnospiraceae↓*	(170)
Polysaccharide from Enteromorpha clathrata	DSS-induced ulcerative colitis in mice	*Parabacteroides Lachnospiraceae NK4A136 Lactobacillus johnsonii Muribaculaceae Parabacteroides* and *Alistipes↑;Akkermansia muciniphila* and *Bacteroides thetaiotaomicron↓*	(171)

"↑" indicates an increase in the abundance of the corresponding intestinal microbiota taxa, and "↓" indicates a decrease in the abundance of the corresponding intestinal microbiota taxa.

## 7 Nanotechnology delivers polysaccharides to treat IBD

We therefore conceptualize polysaccharide nanomedicines as mechanism amplifiers: mucoadhesion and receptor targeting extend residence (boosting barrier repair); pH/ROS/enzyme responsiveness gates on-site release (boosting anti-inflammatory and antioxidant actions); and co-delivery strategies align metabolic support (SCFAs) with immune reprogramming (Tregs↑, M1→M2). The following subsections organize the evidence not by polymer name, but by pathophysiological lever addressed, creating a one-to-one mapping between IBD axes and nanodesign features. Natural polysaccharides are attractive candidates for nanocarrier development in IBD therapy due to their inherent bioactivity, pH responsiveness, gastric stability, susceptibility to colonic microbial degradation, and strong mucoadhesive properties (172–176). By modifying functional groups on their surfaces, polysaccharide-based nanocarriers can be engineered to encapsulate drugs, achieve sustained release, and selectively target specific gut microbial populations (177).

Nanomedicine delivery systems, owing to their nanoscale dimensions and unique structural properties, enhance drug accumulation and retention at target sites, thereby supporting localized therapy (178). The viscosity and intrinsic charge of polysaccharides further enable intimate interactions with the intestinal barrier, prolonging retention within the colon (13, 179). For instance, positively charged nanoparticles adhere to or penetrate negatively charged mucosal surfaces via electrostatic interactions, whereas negatively charged nanoparticles preferentially accumulate in positively charged inflamed tissues, thereby improving lesion targeting. Moreover, polysaccharide-based nanocarriers promote cellular uptake by IECs and immune cells through endocytosis and exocytosis (180). Collectively, these systems improve solubility, intestinal retention, and site-specific drug accumulation, resulting in enhanced therapeutic efficacy and reduced systemic side effects (172, 181).

**Figure 3:**
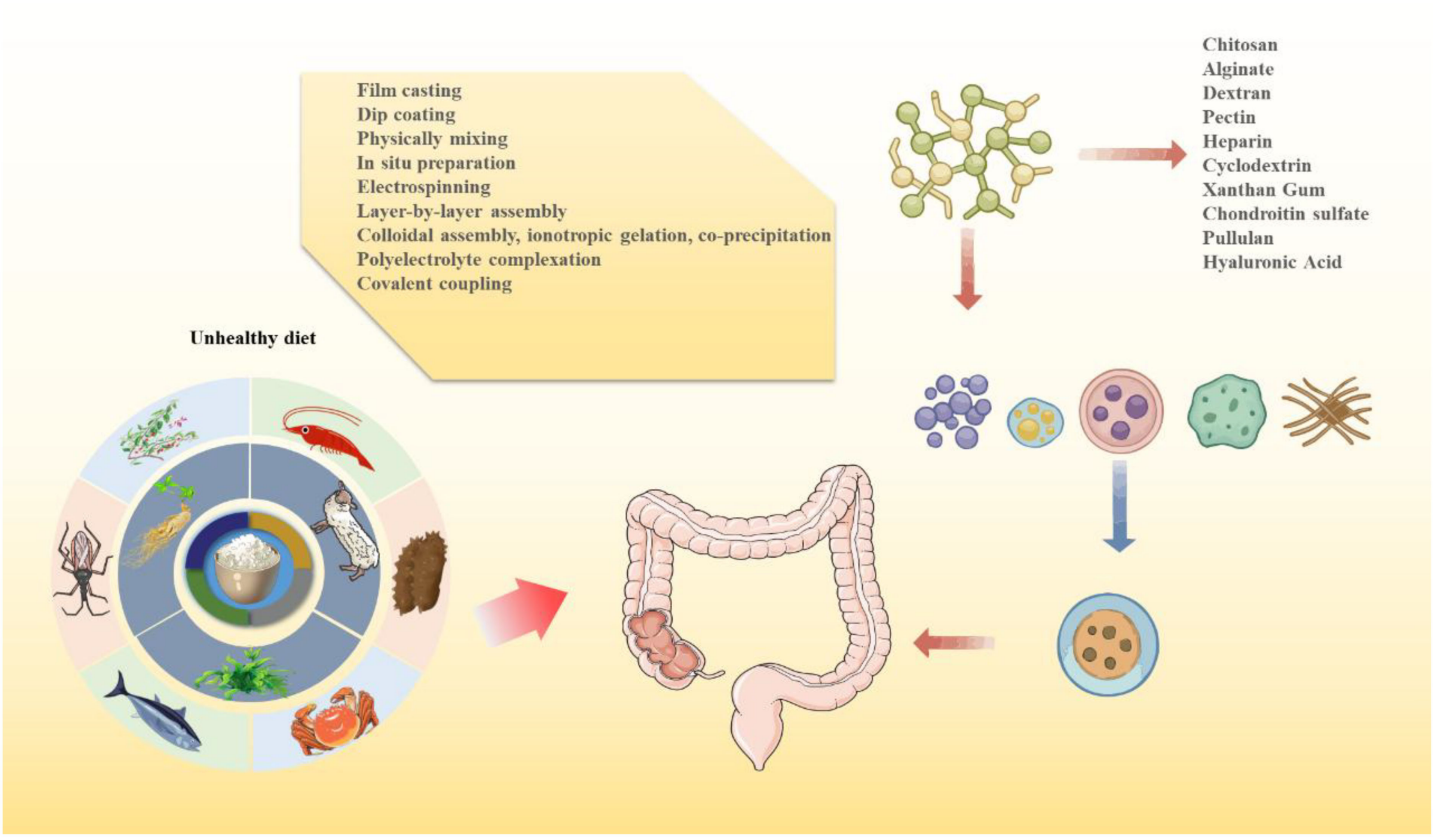
Sources of natural polysaccharides for nanodelivery systems.

Currently, chitosan (CS), alginate (ALG), hyaluronic acid (HA), and *Angelica sinensis* polysaccharide (ASP) are among the most widely studied polysaccharide-based nanocarriers for IBD treatment, owing to their favorable biocompatibility and functionality (18, 19) (**Figure 3**). However, some polysaccharides exhibit high solubility and poor film-forming ability, leading to premature drug release and reduced colonic targeting. To address these limitations, composite nanocarrier systems combining multiple polysaccharides have been developed, effectively overcoming the weaknesses of single-component carriers (182, 183). Despite these advances, challenges remain, including instability in gastric acid and limited colon-targeting efficiency. Moreover, clinical data on dose-response relationships of polysaccharides in IBD remain scarce (131).

### 7.1 Properties of Cs and application of nano-carriers

CS is a cationic natural polysaccharide characterized by its positive charge, mucoadhesive properties, biocompatibility, non-toxicity, and biodegradability (136). Its cationic nature facilitates electrostatic interactions with the negatively charged mucus layer, thereby extending its retention time in the intestinal mucosa (184). As CS resists degradation in the upper gastrointestinal tract, orally administered CS can reach the colon intact, exerting localized therapeutic effects. In a DSS-induced colitis mouse model, CS reduced disease activity, ameliorated histopathological alterations, upregulated tight junction proteins, decreased TNF expression, and modulated gut microbial composition by increasing *Lactobacillus* and *Blautia* populations (185).

CS can also remodel gut microbial communities by increasing the abundance of probiotics such as *Prevotella, Vibrio*, and SCFA-producing taxa (186). Notably, the pH responsiveness of CS further supports its microbiota-regulating effect: Its amino groups impart pH responsiveness, enabling environmentally triggered drug release—it remains stable in the acidic gastric environment (pH 1.2) to avoid premature degradation, and only dissociates in the neutral-to-weakly alkaline colonic environment (pH 6.0-7.0) where it can directly interact with gut microbes and exert probiotic-enriching effects (187, 188). This pH-responsive feature, combined with targeted delivery, enhances therapeutic synergy: For instance, amphiphilic CS nanoparticles self-assembled with quercetin allow targeted intestinal delivery (189).CS hydrogels swell more under acidic than alkaline conditions, making them effective carriers for gastric nutrient release (190). A CS-pectin delivery system has also been developed to encapsulate anthocyanins, protecting them through the gastric environment and ensuring controlled release in the small intestine, thereby improving stability and bioavailability (191).

A dual-responsive nanodelivery system, RH-F/C-NPs, based on CS and fucan, exhibits pH/ROS sensitivity and strong mucoadhesion, making it suitable for ulcerative colitis therapy. This system significantly decreased pathogenic bacteria while increasing beneficial species such as *Lactobacillus* (192). The composite nanosystem achieves stable structure through electrostatic and hydrogen bonding interactions, ensuring efficient colon delivery and microbiota regulation (193).

Due to the strong adhesion between CS and mucins, CS-based nanoparticles persist in the colon, providing sustained drug release. They also restore gut microbial balance by inhibiting TLR4/NF-κB signaling, activating Nrf2/HO-1 antioxidant pathways, repairing epithelial barriers, and re-establishing gut homeostasis. Importantly, RH-F/C-NPs markedly upregulated barrier-associated proteins including occludin, claudin-1, and ZO-1, underscoring their therapeutic potential for epithelial injury repair (194–197).

### 7.2 Characteristics of alginic acid and application of nano-carriers

ALG is a naturally occurring polysaccharide rich in hydroxyl and carboxyl groups, enabling hydrogen bonding, gel formation, mucoadhesion, and enhanced transdermal penetration (198). Due to its excellent biocompatibility, biodegradability, and drug-loading capacity, ALG nanoparticles (NPs) are readily degraded in biological systems, thereby reducing the risk of long-term accumulation (198). Furthermore, ALG possesses intrinsic pH sensitivity, allowing structural modification for controlled release under specific gastrointestinal conditions.

For example, CS-calcium-ALG microparticles have been developed by crosslinking ALG with polymers and calcium through spray drying, encapsulating *Lactobacillus casei* together with inulin rich in fructooligosaccharides (199). This delivery system significantly improved mucosal integrity, promoted vasodilation and glandular development, and reduced inflammatory cell infiltration in colonic tissues. Moreover, *Lactobacillus* counts in treated rats returned to levels comparable to healthy controls (200).

Notably, shifts in the relative proportions of *Lactobacillus* and pathogenic bacteria such as *Escherichia coli* are closely associated with colonic inflammation (201). Enhancing the abundance of *Lactobacillus* may thus restore *high-molecular-weight* equilibrium and rebalance pro- and anti-inflammatory responses in the gut (202).

### 7.3 Properties of HA and application of nano-carriers

HA, a major component of synovial fluid and the extracellular matrix, exhibits notable immunomodulatory activities. It modulates macrophage function, stimulates antimicrobial peptide production, inhibits bacterial proliferation (203), and regulates CD4^+^ T cell responses. Studies have shown that HA protects the intestinal epithelium by reducing inflammation and permeability, thereby preserving barrier integrity. Chemically modified HA formulations, such as biphasic enema suspensions, significantly decreased inflammation and permeability while maintaining mucosal function in murine colitis models (204).

HA-based nanocarriers have been engineered for enhanced colonic targeting. For example, HA-functionalized polymer nanoparticles preferentially accumulate within inflamed intestinal epithelia compared with native HA, forming a protective barrier and strengthening tight junction signaling (204). Conjugation of HA to CS-modified nanoparticles via amide bonding improved targeting efficiency and cellular uptake, while CS-HA combinations synergistically attenuated colitis symptoms in mice. Amphiphilic HA-bilirubin conjugates have also been developed to form HA-bilirubin nanomedicine (HABN) (205). HABN preferentially accumulates in inflamed IECs, restores barrier integrity, and reshapes the gut microbiota, enriching *Akkermansia muciniphila* and *Clostridium* cluster XIV, both critical for gut homeostasis (206). *A. muciniphila* and its outer membrane protein Amuc alleviate inflammation by modulating host immune responses. HABN also increased *Lactobacillus* abundance (207), complementing the butyrate-mediated Treg activation by *Clostridium* cluster XIV (192).

Current evidence suggests that Lactobacillus exerts anti-inflammatory effects in various animal models of colitis (208–210) and in patients with IBD (211–213). Notably, treatment with broad-spectrum oral antibiotics partially diminished the protective efficacy of HABN against DSS-induced colitis, underscoring the role of microbiota in mediating these effects.

HA can also be metabolized by gut probiotics including *Bacteroides*, *Lactobacillus*, and *Bifidobacterium*, which degrade orally administered HA into unsaturated oligosaccharides. These metabolites are further converted into SCFAs, CO_2_, and H_2_ (214), providing nutrients for IECs and reinforcing epithelial defenses (215). SCFAs support epithelial turnover, mucosal growth, and immune regulation. In IBD models, HABN reduced tissue injury, immune infiltration, and peroxidase activity while enhancing colon length and antimicrobial peptide expression (216). Interestingly, the regulatory effects of HA within the gut depend on its molecular weight (217–221). High-molecular-weight HA stabilizes the intestinal mucosa and counteracts immune dysregulation, whereas low-molecular-weight HA enhances metabolic absorption and modulates innate immune responses. Specific HA fragment sizes also exhibit distinct biological activities, playing pivotal roles in inducing immune defense mechanisms within the intestinal epithelium (222, 223).

### 7.4 Characteristics of ASP and application of nano-carriers

Water-soluble polysaccharides can be transformed into amphiphilic polymers through partial dehydrogenation, enabling spontaneous self-assembly in aqueous environments. In such systems, hydrophobic moieties aggregate to form the core, while hydrophilic polysaccharide chains constitute the shell, yielding stable micellar structures (224). The hydrophobic core accommodates hydrophobic drugs via noncovalent interactions, whereas the hydrophilic shell can be chemically modified with responsive groups for controlled or targeted release (225).

ASP, owing to its high solubility, biocompatibility, biodegradability, abundant hydroxyl groups, and modifiability, is an ideal candidate for constructing amphiphilic polymeric micelles with therapeutic potential (226, 227). For instance, cystine dihydrochloride has been used as a crosslinker to synthesize ASP-based nanoparticles encapsulating proanthocyanidins for ulcerative colitis therapy. These nanoparticles were glutathione-sensitive, enabling efficient release in inflamed tissues. However, due to the complexity of the colonic microenvironment, single-responsive systems often fail to ensure precise delivery. To overcome this limitation, dual-responsive ASP nanocarriers, sensitive to both pH and redox conditions, were developed to deliver ginsenoside Rh2 selectively to inflamed colonic sites. This system significantly alleviated colitis symptoms and modulated gut microbial composition (228, 229).

ASP has also been chemically modified with allantoic acid to generate amphiphilic polymers that self-assemble into nanoparticles through carboxyl-mediated interactions. The imidazole group of allantoic acid confers pH sensitivity, promoting rapid degradation in acidic inflammatory sites (230). Similarly, ASP conjugation with α-lipoic acid introduces redox responsiveness via disulfide bonds, enabling degradation under glutathione-rich conditions (231). *In vivo* studies demonstrated that ASP-based nanocarriers enriched beneficial taxa such as *Norank*, *Lactobacillus*, and *Lachnospiraceae*, while reducing harmful genera including *Bacteroides*, *Turicibacter*, and *Ruminococcus*. These Rh2-loaded ASP nanoparticles enhanced SCFA production, particularly acetate, propionate, and butyrate, and upregulated ZO-1 expression in colonic tissues, thereby improving mucosal barrier homeostasis (232). The nanoparticles exhibited dual targeting: passive accumulation in inflamed tissues through the enhanced permeability and retention (EPR) effect, and active targeting via dual responsiveness. Their small particle size also facilitated cellular uptake by IECs and immune cells (e.g., neutrophils, macrophages, and M cells) through endocytosis (172). Collectively, ASP nanocarriers not only enhanced drug bioavailability but also increased anti-inflammatory efficacy in colitis therapy.

### 7.5 Characteristics of rhubarb polysaccharides and application of nano-carriers

Rhubarb polysaccharide (DHP), predominantly extracted from rhubarb, is characterized by its biodegradability, low immunogenicity, and minimal toxicity. Its abundant hydroxyl groups facilitate electrostatic and hydrogen-bond interactions, enabling co-assembly with berberine (BBR) to form BBR-DHP nanoparticles (BD). Studies using DSS-induced colitis models revealed that disease groups exhibited increased *Proteobacteria* and decreased *Firmicutes*. BD treatment restored microbial balance, reducing *Proteobacteria* and enriching *Lactobacillus*, a key probiotic genus within *Firmicutes* that promotes gut homeostasis (233).

*Lactobacillus* contributes to epithelial repair, mucosal defense, and immune regulation. It competitively inhibits pathogen adhesion to IECs, produces antimicrobial metabolites (e.g., lactic, acetic, and propionic acids, bacteriocins, ROS), and strengthens host defenses (234). Notably, *Lactobacillus* abundance was significantly reduced in both DSS and BBR-only groups, but maintained in the DHP and BD groups, consistent with genus- and phylum-level shifts. These findings suggest that the microbiota-modulating effect of BD is primarily attributable to DHP (233). Histological analyses further demonstrated that BD treatment ameliorated colonic injury, restoring crypt architecture, preserving goblet cells, and reducing muscular edema. BD also maintained colon length (~8 cm), comparable to healthy controls. Tight junction proteins occludin and ZO-1, markedly reduced in DSS groups, were restored by BD treatment. Deficient O-glycosylation compromises mucin production and disrupts the mucus barrier, thereby facilitating inflammasome activation (e.g., caspase-1, IL-1, IL-18) and exacerbating inflammation. High-performance liquid chromatography (HPLC) revealed that minor monosaccharides in DHP—such as mannose, xylose, and GalNAc—may promote glycosylation, reduce inflammation, and contribute to therapeutic efficacy (235, 236).

### 7.6 Properties of *Phellodendron amurense* polysaccharides and application of nano-carriers

*Phellodendron amurense* polysaccharide (PIP) has demonstrated the ability to improve the intestinal microenvironment by modulating gut microbiota and enhancing mucosal immunity (237). Given its potent anti-inflammatory and prebiotic properties, researchers developed a PIP-loaded CS-modified poly(lactic-co-glycolic acid) (PLGA) nanoparticle (CS-PIPP). Experimental results showed that CS-PIPP decreased pathogenic taxa while increasing beneficial bacteria such as *Lactobacillus* and *Akkermansia muciniphila*, underscoring its potential as a prebiotic agent (238). Further investigations revealed that both free PIP and CS-PIPP increased *Lactobacillus* and *A. muciniphila* populations. Notably, CS-PIPP more effectively reduced *Escherichia coli* and *Shigella* abundance, thereby limiting pathogen invasion of colonic mucosa and suppressing inflammatory responses. In addition, CS-PIPP enriched probiotic genera such as *Alloprevotella* while reducing harmful taxa including *Romboutsia*, often elevated in ulcerative colitis (239, 240).

CS-PIPP also exerted immunomodulatory effects. It enhanced IL-10 secretion, inhibited M1 macrophage polarization, and preserved tight junction proteins (ZO-1 and occludin), thereby maintaining barrier integrity. Regulation of SCFA production may represent an additional protective mechanism. Collectively, these findings highlight CS-PIPP as a synbiotic nanocarrier with multifaceted roles in protecting against IBD through reshaping the microbiota, strengthening the barrier, and modulating immune responses (241–243).

### 7.7 Characteristics of Eucommia ulmoides polysaccharide and application of nano-carriers

Eucommia ulmoides polysaccharide (EUP) refers to a group of sugars extracted from the leaves and roots of Eucommia ulmoides. Previous studies have shown that EUP possesses anti-inflammatory, antioxidant, and immunomodulatory properties (244, 245). Selenium nanoparticles (SeNPs) are known for their excellent biological activity in IBD therapy. In this context, EUP was used as a surface modifier to prepare EUP-SeNPs with an approximate size of 170 nm. Oral administration of EUP-SeNPs effectively counteracted DSS-induced reductions in beneficial bacteria such as Actinomycetes, DNA, Rikenellaceae, and Muribaculaceae. Concurrently, they decreased the abundances of pathogenic bacteria including Campylobacter, Escherichia coli, Vibrio, Desulfobacter, and Ruminococcus. These findings align with previous studies (246, 247), suggesting that EUP-SeNPs can mitigate colonic injury by modulating the gut microbiota, enhancing beneficial taxa, and suppressing harmful populations (248).

Mucins in the intestinal mucus layer act as a primary defense by preventing pathogen infiltration (249, 250). Studies revealed that EUP-SeNPs improve the expression of tight junction proteins by reducing inflammatory cell infiltration and intestinal permeability, increasing goblet cell numbers and mucin secretion, regulating IEC apoptosis and proliferation, and modulating inflammatory cytokines, collectively ameliorating DSS-induced colonic damage. Additionally, EUP-SeNPs inhibited activation of the TLR4/NF-κB signaling pathway. Maintaining redox balance is critical for overall health, with multiple indicators used to evaluate colonic oxidative status. Oral administration of EUP-SeNPs was found to significantly enhance colonic antioxidant capacity and attenuate the severity of DSS-induced colitis, underscoring their potential as a multifunctional therapeutic strategy for IBD (248).

### 7.8 Other polysaccharides

Polysaccharides from various natural sources, including *Codonopsis pilosula*, *Dendrobium officinale*, *LBP*, and *Tremella fuciformis*, have also shown therapeutic promise against IBD (**Table 2**).

**Table 2:** BD nanodesign mechanisms, pathological axes, and therapeutic outcomes.

Nanodesign mechanism	Corresponding IBD pathological axis	Representative materials/systems	Key therapeutic outcomes
Mucoadhesion & Retention	Barrier disruption (mucus thinning, loss of tight junction proteins)	CS nanoparticles; HA nanoparticles	Enhanced adhesion, reduced DAI, increased colon length, upregulation of ZO-1/occludin, thicker mucus layer
pH-Responsive Release	Microbiota dysbiosis+acidic inflamed environment	pH-sensitive CS-based systems	Increased drug concentration at inflamed sites, reduction of pathogens, enrichment of probiotics
ROS/Redox-Responsive Release	Oxidative stress (ROS accumulation, GSH upregulation)	ROS - or redox - sensitive polysaccharide systems (e.g., CS-fucan, ASP-LA)	Decreased ROS and MDA, activation of Nrf2/HO-1 pathway, reduced inflammatory cytokines
Enzyme-Responsive Degradation	Microbiota dysbiosis (overexpression of microbial enzymes)	Polysaccharide nanoparticles degradable by bacterial enzymes	Enrichment of beneficial bacteria, elevated SCFAs, alleviation of inflammation
Receptor-Mediated Targeting	Immune dysregulation (Treg depletion, NF-κB activation)	HA-modified nanomedicines targeting CD44; β-glucan-based ligands targeting Dectin-1	Increased Treg levels, upregulated IL-10, M1→M2 macrophage polarization, inhibition of NF-κB
Co-Delivery & Synergy	Combined pathological axes (microbiota, barrier, immunity, oxidative stress)	Dual-delivery systems combining polysaccharides with small molecules or inorganic nanoparticles	Decreased pathogens, enrichment of probiotics, restoration of tight junction proteins, enhanced antioxidant enzymes, suppression of TLR4/NF-κB signaling

*Codonopsis pilosula* polysaccharides (CPP) help maintain gut homeostasis by sustaining *Lactobacillus* abundance and reducing *Escherichia-Shigella* populations, thereby restoring microbial balance (251). *Dendrobium officinale* polysaccharides (DOP) significantly increased microbial diversity and improved the relative abundances of *Firmicutes* and *Bacteroidetes* in colitis models. DOP also suppressed harmful taxa such as *Proteobacteria* and reduced inflammation, oxidative stress, and apoptosis, ultimately enhancing barrier integrity by upregulating ZO-1 and occludin expression (252). LBPs improved microbial composition by elevating *Lactobacillus* and *Bifidobacterium*, restoring microbial diversity, and strengthening mucosal defenses. LBP supplementation increased SCFA levels, reduced colonic inflammation, and upregulated tight junction proteins, thereby ameliorating colitis pathology (253, 254). *Tremella fuciformis* polysaccharides (TFP) exhibited strong microbiota-regulating activity by increasing SCFA-producing bacteria and *Lactobacillus*, while simultaneously enhancing tight junction protein expression and reducing oxidative stress. These effects collectively protected epithelial integrity and alleviated colitis (255). In addition, selenium nanoparticles prepared with seaweed polysaccharides demonstrated anti-inflammatory efficacy by inhibiting NF-κB activation, preserving intestinal barrier integrity, and reducing inflammation in colitis models (256).

## 8 Challenges and prospects

This review has systematically analyzed the core pathological mechanisms underlying IBD, including gut microbiota dysbiosis, impaired intestinal barrier function, immune dysregulation, and oxidative stress. Natural polysaccharides, derived from diverse sources and exhibiting distinct structural characteristics, demonstrate strong therapeutic potential in modulating these pathways. Their dual mechanisms—microbiota regulation and barrier enhancement—include stimulation of beneficial bacteria such as *Lactobacillus* and *Bifidobacterium*, elevation of SCFA production (e.g., butyrate as an epithelial energy source, acetate for Treg differentiation), inhibition of TLR4/NF-κB inflammatory signaling, and activation of the Nrf2/HO-1 antioxidant pathway. Moreover, the context-dependent roles of key microbes, such as *Akkermansia muciniphila*, highlight their ability to maintain barrier integrity in health but exacerbate inflammation under pathological conditions.

Polysaccharide-based nanocarriers—including CS, ALG, HA, and *ASP*—further enhance therapeutic efficacy by enabling targeted, responsive, and sustained drug delivery. Smart designs, such as pH/ROS dual-sensitive RH-F/C-NPs and composite carriers like CS-PIPP, combine prebiotic activity, controlled release, and mucoadhesion, achieving superior probiotic enrichment and pathogen suppression compared with single-component systems. Recent advances have also underscored the importance of polysaccharide structure-activity relationships. For instance, low-molecular-weight fucoidan (2.56 kDa) and konjac glucomannan (KGM2, 7413 Da) exhibit enhanced anti-inflammatory activity due to improved microbial fermentation into SCFAs, while branched polysaccharides such as SHPS-1 exert effects via specific glycosidic linkages (257).

Despite these promising developments, challenges remain. Structural complexity, variability in extraction and purification, and difficulties in achieving reproducible formulations hinder translational application. Furthermore, polysaccharide bioactivity is strongly influenced by molecular weight, branching degree, and monosaccharide composition (258, 259). Although low-molecular-weight fractions often show superior bioactivity (260–262), results remain inconsistent. Future efforts should focus on clarifying structure-activity relationships, designing multi-responsive nanocarriers for precise release, and extending applications to biomacromolecule delivery systems such as vaccines and nucleic acids. In conclusion, polysaccharides and their nanoformulations represent highly promising therapeutic strategies for restoring microbial homeostasis, reinforcing mucosal barriers, and attenuating intestinal inflammation in IBD.
